# Nitric oxide gas phase release in human small airway epithelial cells

**DOI:** 10.1186/1465-9921-10-3

**Published:** 2009-01-19

**Authors:** Jingjing Jiang, Nikita Malavia, Vinod Suresh, Steven C George

**Affiliations:** 1 Department of Biomedical Engineering, University of California Irvine, Irvine, CA, USA; 2 Department of Chemical Engineering and Material Science, University of California Irvine, Irvine, USA

## Abstract

**Background:**

Asthma is a chronic airway inflammatory disease characterized by an imbalance in both Th1 and Th2 cytokines. Exhaled nitric oxide (NO) is elevated in asthma, and is a potentially useful non-invasive marker of airway inflammation. However, the origin and underlying mechanisms of intersubject variability of exhaled NO are not yet fully understood. We have previously described NO gas phase release from normal human bronchial epithelial cells (NHBEs, tracheal origin). However, smaller airways are the major site of morbidity in asthma. We hypothesized that IL-13 or cytomix (IL-1β, TNF-α, and IFN-γ) stimulation of differentiated small airway epithelial cells (SAECs, generation 10–12) and A549 cells (model cell line of alveolar type II cells) in culture would enhance NO gas phase release.

**Methods:**

Confluent monolayers of SAECs and A549 cells were cultured in Transwell plates and SAECs were allowed to differentiate into ciliated and mucus producing cells at an air-liquid interface. The cells were then stimulated with IL-13 (10 ng/mL) or cytomix (10 ng/mL for each cytokine). Gas phase NO release in the headspace air over the cells was measured for 48 hours using a chemiluminescence analyzer.

**Results:**

In contrast to our previous result in NHBE, baseline NO release from SAECs and A549 is negligible. However, NO release is significantly increased by cytomix (0.51 ± 0.18 and 0.29 ± 0.20 pl^.^s^-1.^cm^-2^, respectively) reaching a peak at approximately 10 hours. iNOS protein expression increases in a consistent pattern both temporally and in magnitude. In contrast, IL-13 only modestly increases NO release in SAECs reaching a peak (0.06 ± 0.03 pl^.^s^-1.^cm^-2^) more slowly (30 to 48 hours), and does not alter NO release in A549 cells.

**Conclusion:**

We conclude that the airway epithelium is a probable source of NO in the exhaled breath, and intersubject variability may be due, in part, to variability in the type (Th1 vs Th2) and location (large vs small airway) of inflammation.

## Background

Asthma is a chronic inflammatory disease characterized by airway hyperresponsiveness and variable airflow obstruction. Activation of eosinophils, T lymphocytes, neutrophils and macrophages are all involved in airway inflammation, which can trigger the release of mediators and cytokines that contribute to the clinical syndrome of asthma. Among these, IL-13, a cytokine derived from type 2 lymphocytes (Th2), has been proposed to play a major role in the development of atopic asthma [1]. In addition, cytokines secreted by type 1 lymphocytes (Th1) and macrophages, including IL-1β, TNF-α, and IFN-γ, may be increased in asthma subjects and contribute to the inflammatory process [2].

Inflammation in asthma occurs throughout the airway tree. Increasing evidence demonstrates that inflammation of smaller airways (diameter < 2 mm) is significant, and contributes to airways hyperresponsiveness, nocturnal asthma, and asthma exacerbations [3]. Because of the relative inaccessibility of small airways, examination of the inflammatory process is mainly limited to post-mortem or bronchial biopsy analysis while dynamic *in vivo *assessment remains limited to estimates of small airway and alveolar concentration based on exhaled NO levels and mathematical models [4].

The fractional concentration of exhaled nitric oxide (F_ENO_) is a potential biomarker of inflammation in asthma, which may be useful in both diagnosis and treatment. F_ENO _increases in untreated asthma, and decreases with corticosteroid treatment [5], but its clinical utility remains limited mainly due to significant intersubject variability. The exhaled NO signal can be partitioned into airway and alveolar components by measuring exhaled NO at multiple flows and applying mathematical models. Studies suggest that alveolar NO is a measure of inflammation in the distal lung [6]. Increased iNOS (inducible nitric oxide synthase, NOSII) expression in airway epithelial cells has been proposed to be the main source of exhaled NO [7]. Our recent study directly measured gas phase NO release in primary normal human bronchial epithelial cells (NHBEs) of tracheal origin, and demonstrated significant intersubject variability in response to IL-13 [8]. NO production has not been directly examined in airway epithelial cells of small airway origin.

We hypothesized that primary human small airway epithelial cells (SAECs) would produce detectable NO gas phase release following stimulation by IL-13 and cytomix (IL-1β, TNF-α, and IFN-γ). A549 cells (human lung epithelial carcinoma cell line, model of human alveolar epithelial type II cells), which have been used extensively for the investigation of NO metabolism [9-13], were also utilized in this study. Our results demonstrate that cytomix rapidly (<10 hours) upregulates iNOS expression and gas phase NO release in both SAECs and A549. In contrast, IL-13 did not induce significant NO release in A549 cells, and caused only a modest, and delayed (30 to 48 hours), response from SAECs. This suggests the small airway epithelium is a likely source of exhaled NO in inflammatory diseases such as asthma, and responds to inflammatory stimuli in a pattern distinct from epithelial cells of large airway origin [8].

## Materials and methods

### Cell culture

Cryopreserved passage 1 small airway epithelial cells (SAECs) from 3 different donors (donor1: 4F0715, donor 2: 6F3342, donor 3: 6F3426) were purchased from Lonza (formerly Cambrex, Walkersville, MD) and grown on T-75 cm^2 ^flasks (Corning, Fisher) in a 37°C, 5% CO_2_/95% air incubator in small airway epithelial basal medium (SABM) supplemented with growth factors supplied in the SAGM SingleQuot^® ^kit (Lonza). At passage 3, cells were trypsinized and seeded onto Costar Transwells^® ^inserts with 0.4 μm pore size (Corning, Fisher) at a density of 2.0 × 10^5^cells/well in media comprised of 50% SABM and 50% DMEM-F12 low glucose (Invitrogen, Carlsbad, CA) with the same final concentration of supplements as used for flasks, and additional retinoic acid (50 nM). Medium was applied both apically and basally until the cells reached confluence, at which time an air-liquid interface (ALI) was established for 7 days to achieve mucociliary differentiation. The media was changed every other day, and transepithelial electrical resistance (TER) was measured by Millicell-ERS (Millipore, Bedford, MA) at room temperature every other day starting from the second day of ALI.

A549 cells were obtained from the American Type Culture Collection (ATCC, Rockville, MD) and were grown on T-75 cm^2 ^flasks (Corning, Fisher) in a 37°C, 5% CO_2_/95% air incubator in F12-k medium (ATCC, Rockville, MD) containing 10% fetal bovine serum (Mediatech, VA), penicillin (100 U/ml, Mediatech, VA) and streptomycin (100 μg/ml, Mediatech, VA). When subconfluent, cells were trypsinized and seeded onto Costar Transwells^® ^inserts with 0.4 μm pore size (Corning, Fisher) at a density of 2.0 × 10^5 ^cells/well. Once the cells reached confluence, they were shifted to ALI culture for 2 days.

### Immunofluoresent staining for cell differentiation markers

After 7 days of ALI, SAECs were fixed in 4% paraformaldehyde for 30 mins and then permeabilized with 1% Triton X-100 in PBS for 20 min. Nonspecific immunogloblin binding was blocked by incubation with 5% normal goat serum in PBS for 1 h at room temperature. Mouse monoclonal antibodies against ZO-1 (1:250 dilution; Zymed Laboratories, South San Francisco, CA), β-tubulin IV (1:1000 dilution; Sigma, St. Louis, MO), and mucin 5AC (MUC5AC, 1:1000 dilution; Neo Markers, Fremont, CA) were diluted in PBS with 5% goat serum and incubated at 4°C overnight. The samples were washed and then incubated in Alexa Fluor 488 anti-mouse secondary antibody (Molecular Probes, Eugene, OR) diluted 1:500 in PBS with 5% goat serum for 2 hours at 4°C. Cell nuclei were stained with 4', 6-diamidino-2-phenylindole dihydrocholride hydrate (1 μg/ml, DAPI, Sigma) in PBS for 5 minutes. Fluorescence images were obtained using a Nikon Eclipse E800 epifluorescence microscope.

### Stimulation of SAECs and A549 with cytomix or IL-13

Culture medium was changed 24 hours prior to the experiment. On the day of the experiment (t = 0 hour), cytomix (TNF-α, IL-1β, IFN-γ, R&D Systems, Minneapolis, MN) was added to fresh culture medium to achieve a final concentration of 10 ng/ml each. For the IL-13 group, IL-13 (R&D Systems, Minneapolis, MN) was added to the medium to achieve a final concentration of 10 ng/ml. Concentrations of cytokines were based on previous reports to achieve iNOS expression without compromising cell viability [8,10,14]. 24 hours after the experiment started, 30 μM iNOS inhibitor L-NIL (N6-(1-iminoethyl)-L-lysinem, Cayman chemical, Ann Arbor, MI) was added to the cytomix treated culture medium in some experiments. The total duration of each experiment was 48 hours.

### Gas phase NO measurement and NO flux calculation

12-well Transwell^® ^plates were fitted with modified lids and edges were sealed with Parafilm M (Menasha, WI) to form a gas tight enclosure. Holes were drilled on the top surface of the lids. The plates were placed in a 37°C, 5% CO_2_/95% air incubator and one of the holes was connected to the inlet of a chemiluminescent nitric oxide analyzer (NOA 280, Sievers, Boulder, CO) via a flow meter. A constant flow (Q) of 40 ml/s was used to ensure an accurate reading from the NOA. The rate of NO gas transport (flux) from the cells to the airstream is independent of the gas flow due to the low water solubility of NO [4]. Real time NO data at different time points from the NOA was collected for further analysis. NO flux was calculated as previously described [8]. Multiple measurements were performed between 7 to 21 days following air-liquid interface. In brief, real time NO reaches a plateau value, Cp (ppb), representing the steady state NO release into the gas phase after the washout of accumulated NO from the headspace. Steady state NO concentrations were determined by fitting an exponential form to the smoothed transient response and the NO flux was calculated as F = QC_p_/A_s _(pl^.^s^-1.^cm^-2^) based on the surface area, A_s_, of the Transwells.

### Total nitrite+nitrate assay

Total nitrite+nitrate in the culture medium was measured by a Griess assay kit (Cayman Chemical, Ann Arbor, MI) according to the manufacturer's instructions. Nitrate in the sample medium and standards were converted to nitrite by nitrate reductase, and Griess reagent was added in the 96 well plate. Absorbance was determined at 540 nm. The concentration of total nitrite+nitrate was calculated according to a standard curve of known nitrite concentrations.

### Western Blotting

At each time point after NO gas phase measurement, protein was extracted using RIPA buffer and quantified using the Bradford assay (BioRad). Samples (40 μg equal protein) were subjected to 7.5% SDS-PAGE and transferred to a Polyvinylidene fluoride (PVDF) membrane (Millipore, Bedford, MA). The blots were probed with monoclonal mouse anti-iNOS antibody (Research and Development Antibodies, Las Vega, NV) using 1:1000 dilution in TBST with 2% goat serum and subsequently incubated with horseradish peroxidase (HRP) conjugated secondary antibodies (1:10,000, Santa Cruz biotechnology, Santa Cruz, CA). The proteins were visualized using an enhanced chemiluminescence system (Amersham Biosciences and Biorad Imaging system). The blots were also probed with mouse monoclonal anti-β-actin (Abcam, Cambridge, MA) as a loading control.

### Reverse Transcription and PCR

At the end of experiment (t = 48 hours), RNA was collected from control, cytomix treated and IL-13 treated groups. Total RNA was isolated using NucleoSpin^® ^RNA II kit (Macherey-Nagel, PA) and quantified by Quant-iT™ RiboGreen^® ^RNA assay kit (Invitrogen, Carlsbad, CA). Reverse transcription was carried out using TaqMan^® ^reagents (Applied Biosystems, Foster City, CA). Gene expression of the NOS isoforms was probed via PCR using the primers 5'-GCCTCGCTCTGGAAAGA-3', 3'-TTCCAACAGACGTACCT-5' (iNOS); 5'-AGGACAGACGGCAAGCACGA-3', 3'-GGTGGCGGAGTGATGGTCAA-5' (nNOS); 5'-CAGTGTCCAACATGCTGCTGGAAATTG-3', 3'-CCGTAGTGGTCCTTCTTCTGGAAAT-5' (eNOS). Ribosomal 18S RNA (Applied Biosystems, Foster City, CA) was used as an internal standard.

### Statistics

Experiments were performed using three different SAEC donors with multiple repeats of each donor. Data are presented as Mean ± SD and statistical significance was tested using a two-tailed Student's *t-*test; *p *values less than 0.05 were considered significant.

## Results

### Small airway epithelial cells differentiation

**Figure 1:**
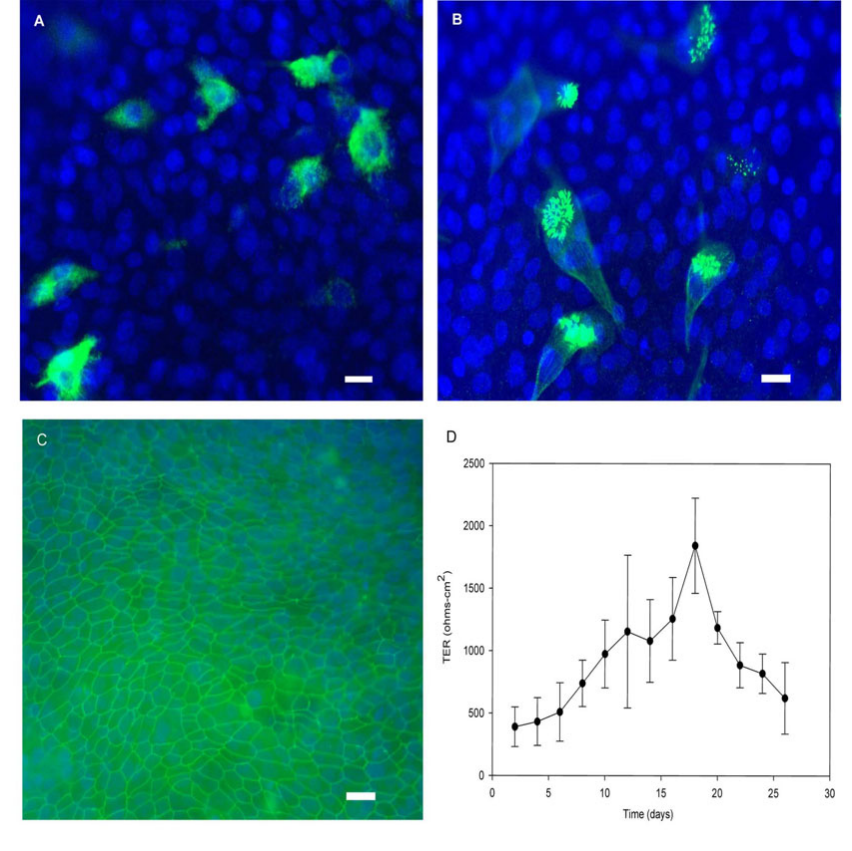
**Phenotypic markers of human small airway epithelial cells (SAECs) cultured at an air-liquid interface**. Day 0 represents the first day of air-liquid interface which is generally 48 hours after seeding on the membrane, and all representative images were taken at day 7. A: Immunofluorescence imaging of MUC5AC (green) demonstrating the presence of mucous granules. B: Immunofluorescence imaging of β-tubulin IV (green) demonstrates the presence of ciliated cells. C: Immunofluorescence imaging of zonula occludens-1 (ZO-1) (green), a key protein present in the intercellular tight junctions. Cell nuclei were counterstained blue by DAPI. D: Transepithelial electrical resistance (TER) was measured every other day from 2 days of ALI. TER data represent the mean response from 2 different donors (12 monolayers from each donor). Scale bar: 10 μm.

Immunofluorescent staining for MUC5AC, β – Tubulin IV and ZO-1 was detected after 7 days of ALI. A small number of SAECs expressed MUC5AC (Fig. 1A), a marker for mucous differentiation and β – Tubulin IV (Fig. 1B), a marker for ciliary differentiation. ZO-1 (Fig. 1C) staining showed a highly organized, mature cell-cell junction. TER climbed to 700 ohms-cm^2 ^after 7 days of ALI and gradually reached a peak of approximately 1500 ohms-cm^2 ^following approximately 18 days of ALI (Fig. 1D). TER data represent the mean response from 2 different donors. These phenotypic markers were stable in culture for more than 2 weeks.

### Cytomix and IL-13 induces NO releases into gas phase and total nitrate formation in SAECs and A549

**Figure 2:**
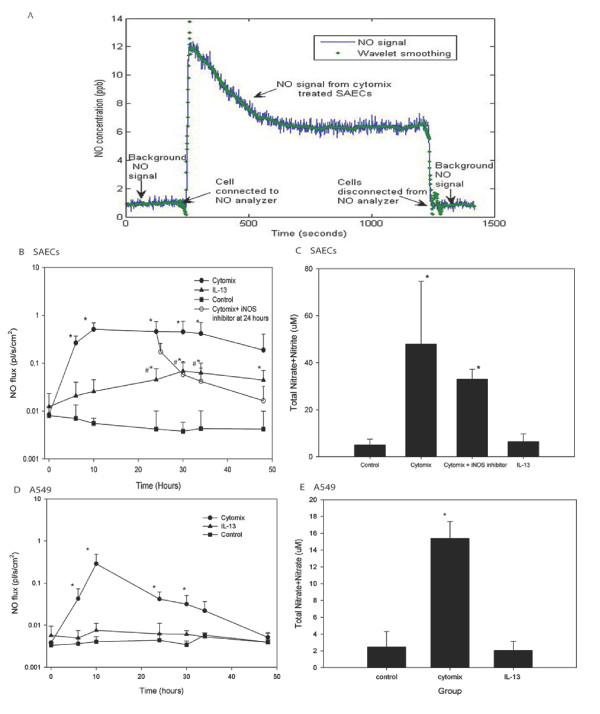
**Gas phase NO release from stimulated SAECs/A549 cells and total nitrate/nitrite in culture medium**. Data is the mean values from 3 donors. A: Representative real-time NO signal from the NO analyzer. Raw analyzer data was smoothed using a wavelet transformation, and the steady state value determined from an exponential fit as previously described [8]. B: Cytomix or IL-13 was added to the SAEC culture medium at t = 0. Gas phase NO concentration and was measured at different times (0, 6, 10, 24, 30, 34 hours) up to 48 hours, and the NO steady state flux was calculated as described in the Methods. Very low level of basal NO flux was detected from SAECs (n = 6, data from 6 measurements). NO flux was increased by cytomix treatment within 6 hours and reached a peak at 10 hours (n = 10 or 11). At t = 24 hours, 30 μM iNOS inhibitor L-NIL was added to the culture medium in some experiments leading to a significant reduction of NO flux (n = 4). IL-13 induced modest increase in NO flux in SAECs (n = 8 or 9, *P < 0.05 compared to control group, #P < 0.05 compared to cytomix stimulated group). C: Total nitrite+nitrate content in SAECs culture medium after 48 hours exposure to either cytomix or IL-13 was measured (n = 10, *P < 0.05 compared to control group). D: Cytomix or IL-13 was added to A549 cell culture medium at t = 0. NO flux was increased by cytomix treatment within 6 hours and reached a peak at 10 hours. IL-13 did not alter the NO flux (n = 4, *P < 0.05 compared to control group). E: Total nitrite+nitrate content in A549 cell culture medium after 48 hours exposure to either cytomix or IL-13 was measured (n = 8, *P < 0.05 compared to control group).

NO gas phase concentration in control, cytomix-treated or IL-13-treated cells was measured at different time points up to 48 hours and the NO flux was calculated. Basal level of NO gas phase release was negligible in both types of cells. Cytomix induced significant NO release at 6 hours and the peak NO flux was observed on average at 10 hours after stimulation (0.51 ± 0.18 and 0.29 ± 0.20 pl^.^s^-1.^cm^2 ^for SAECs and A549 cells respectively) (Fig. 2B, D). Addition of iNOS inhibitor at 24 hours rapidly and significantly reduced the NO flux (Fig. 2B). In contrast, IL-13 induces a modest NO release from SAECs between 30 to 48 hours with a peak of 0.06 ± 0.03 pl^.^s^-1.^cm^-2^, and does not induce NO release from A549 cells (Fig. 2B, D). No significant relation was found between gas phase NO level and days of air-liquid interface (range 7–21 days). No significant variability in NO production was observed among the three SAEC donors.

Total nitrite+nitrate content in the culture medium is another index of total NO production. In SAECs and A549 cells, the total nitrite+nitrate content in the culture medium of the cytomix treated group was significantly higher from control and IL-13 treated cells (Fig. 2C, E) while the total nitrite+nitrate content of in the culture medium of the IL-13 treated group was not significantly different from control cells (Fig. 2C, E). Addition of iNOS inhibitor at 24 hours to cytomix treated SAECs resulted in less total nitrite+nitrate content in the medium than the uninhibited group (Fig. 2C).

### NOS gene and protein expression in SAECs and A549

**Figure 3:**
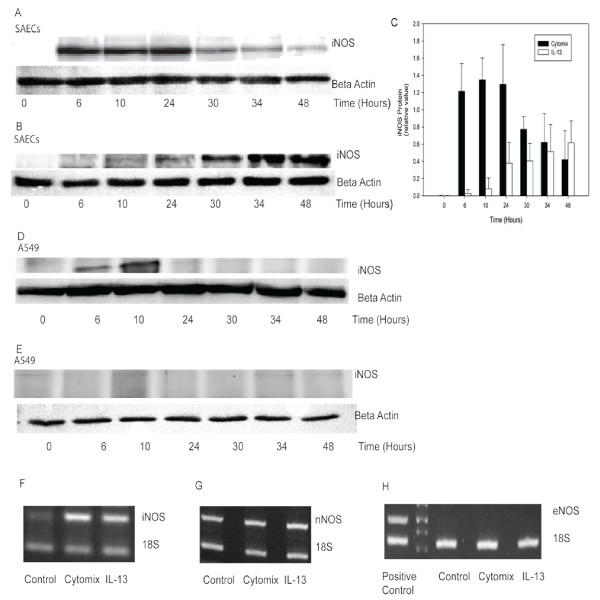
**NOS gene and protein expression in cytomix or IL-13 stimulated SAECs and A549 cells**. A: Cytomix upregulated iNOS protein expression in a pattern consistent with NO flux in SAECs. B: IL-13 steadily enhanced iNOS protein expression from 10 hours to 48 hours in SAECs. C: Densitometry analysis of iNOS protein expression in SAECs normalized by β-actin (n = 3). D: Cytomix induced iNOS protein expressions in A549 cells within 6 hours, reaching a peak at 10 hours. E: IL-13 did not enhance iNOS protein expression within 48 hours in A549 cells. F: Cytomix and IL-13 enhance iNOS mRNA expression after 48 hours exposure in SAECs. G: nNOS mRNA was expressed at baseline in SAEC, but was not altered by cytomix or IL-13 simulation. H: eNOS mRNA was not present in SAECs under basal or cytokine-stimulated conditions.

Unstimulated SAECs and A549 cells did not express detectable iNOS protein by Western blot, but cytomix induced iNOS protein expression in a pattern consistent with NO gas phase release both temporally and in magnitude (Fig. 3A, C and 3D). IL-13 steadily induced iNOS protein expression from 10 hours to 48 hours in SAECs (Fig. 3B), and did not alter iNOS protein expression in A549 (Fig. 3E). iNOS, eNOS and nNOS RT-PCR were performed to investigate the expression of NOS isoforms. A very low level of iNOS mRNA was detected in the control group of SAECs. At t = 48 hours, both cytomix and IL-13 demonstrated elevated iNOS mRNA expression (Fig. 3F). nNOS mRNA was expressed in the control group, but was not upregulated by cytomix or IL-13 simulation (Fig, 3G). eNOS mRNA was not present in SAECs under any conditions (Fig. 3H).

## Discussion

Small airway (airway diameter < 1–2 mm) inflammation is thought to play a critical role in the pathogenesis of asthma including airways hyper-responsiveness, spontaneous exacerbations of symptoms, and tissue remodeling [3]. Non-invasive markers of inflammation, such as NO gas in the exhaled breath, could assist in the management of airway inflammation, but the anatomical source remains unclear. Our study demonstrates that small airway epithelial cells can be differentiated at an air-liquid interface to express markers such as mucin and cilia. The differentiated epithelium produces a very small, but detectable, amount of NO gas at baseline. However, the production is significantly increased, due to the upregulation of iNOS, following exposure to soluble inflammatory mediators, most notably a combination of IL-1β, TNF-α and IFN-γ. As such, iNOS in the small airway epithelium is a probable source of NO in the exhaled breath of asthma.

Bronchioles are generally < 1 mm in diameter, are devoid of cartilage, and are lined with cuboidal epithelial cells. Ciliated cells and mucous producing goblet cells are present, but are less abundant than in larger airways [15]. SAECs are sourced from 1 mm diameter airways (approximately generation 10–12) of normal human subjects, in contrast to normal human bronchial epithelial cells (NHBEs, Lonza) which arise from the trachea (generation 0). Our results demonstrate that SAECs can be cultured at an air-liquid interface for seven days and express mature differentiated markers such as β-tubulin and MUC5AC, markers of cilia and goblet cells. In addition, the cells form a confluent monolayer with a transepithelial electrical resistance (TER) that is similar in magnitude to the NHBEs. The markers of differentiation were detectable as early as 5 days of air-liquid interface, and no significant difference was observed between 7 days and 14 days differentiation (data not shown).

The airway epithelium has been proposed as a major source of NO in the exhaled breath, and our previous work demonstrated that epithelial cells of tracheal origin can produce NO gas at levels which are consistent with that observed in the exhaled breath of healthy subjects. However, asthma is thought to be an inflammatory disease of the smaller airways. The phenotypic features of the airway epithelium change with increasing airway generation. For example, as airway generation increases (smaller diameter airways), the epithelium becomes less columnar (flatter) in shape, expresses less cilia, and produces less mucus [15]. These changes reflect the transition from a barrier function to trap and remove particulates in the inspired air to the gas exchange function of the alveolar region. Our previous results demonstrated significant NO production and release from NHBE at baseline and in response to IL-13 [8]; hence, our results suggest that NO metabolism may also be different in large and small airway epithelium.

At baseline, SAECs released 0.005 ± 0.002 pl^.^s^-1.^cm^-2^(Figure 2B) into the gas phase which is approximately an order of magnitude less than the basal NO release from NHBEs (0.05 ± 0.03 pl^.^s^-1.^cm^-2^) [8]. However, if the increase in surface area with increasing generation number is considered (surface area of adult trachea is ~70 cm^2^, and that of generation 10 is approximately 300 cm^2^), the contribution from these regions to exhaled NO would be of the same order of magnitude (3.5 pl^.^s^-1 ^for the trachea and 1.5 pl^.^s^-1 ^for generation 10). This is consistent with previous reports demonstrating that the main bronchus and trachea contribute approximately 50% of the exhaled NO in normal subjects [16]. Similar to NHBEs [8] and other primary epithelial cells [14], unstimulated SAECs express mRNA for nNOS (NOSI); hence, baseline production is likely due to constitutive expression of nNOS as iNOS protein and eNOS (NOS III) mRNA were not detectable.

Several previous studies have demonstrated that cytomix can induce iNOS expression and nitrite release in A549 cells [9,10], and a combination of TNF-α and IFN-γ increases nitrite content in the medium of NHBEs [17]. Cytomix has also been reported to markedly stimulate iNOS expression in other cells such as intestinal cells [18] and hepatocytes [19]. Our results demonstrated a similar effect of cytomix on iNOS expression and NO release in SAECs and A549 cells. Stimulated by cytomix, SAECs and A549 cells increase gas phase NO release rapidly reaching a peak by 10 hours. The increase correlates temporally with an increase in iNOS protein, and is matched by an increase in total nitrite+nitrate in the media 48 hours after stimulation. The peak NO release from A549 cells and SAECs is similar, but the production is more sustained in SAECs. By 48 hours, the NO release from A549 is back to baseline. The smaller total NO production over the 48 hour time window for A549 cells is reflected in less total nitrite+nitrate (15.5 μM compared to 45 μM). The observation that iNOS protein levels peak at the same time as NO gas release, combined with the elimination of NO release following iNOS inhibition, suggests a direction relationship between iNOS protein level and NO release.

Our previous study in NHBEs demonstrated that 10 ng/ml IL-13 leads to a significant increase in NO flux at 10 hours, and a maximum flux (7.4 pl^.^s^-1.^cm^-2^) at 24 hours [8]. Another recent study also demonstrated that IL-13 can increase iNOS mRNA expression as early as 5 hours after stimulation in primary human airway epithelial cells [14] obtained from bronchial brushings of larger airways. We found that 10 ng/ml IL-13 only modestly and slowly increases NO release from SAECs reaching a peak (0.06 pl^.^s^-1.^cm^-2^) around 30 to 48 hours, which is approximately an order of magnitude smaller and 24 hours slower compared to NHBEs. Our results confirm the ability of IL-13 to enhance NO release and iNOS expression in airway epithelium, and show a pattern in small airway epithelial cells that is distinct from A549 cells. Consistent with previous studies, IL-13 does not impact nitrite production [20] or NO gas release from A549 cells.

The distinct pattern of SAECs in response to cytomix and IL-13 and the difference between SAECs, A549 cells, and NHBEs in response to inflammatory cytokines may assist in the interpretation of the exhaled NO signal. The inflammatory response of asthma is critical to disease progression and therapy. One theory of asthmatic inflammation describes an imbalance in the Th1 and Th2 lymphocyte and cytokine profile. The former produce primarily IL-2 and IFN-γ, and are generally responsible for mounting an immune response to infection. The later produce IL-4, IL-5, IL-6, IL-9, and IL-13 and primarily account for mounting the allergic response. In some asthmatic phenotypes, there may be an imbalance favoring the Th2 response. This is supported by numerous studies showing elevated Th2-type cytokines in the peripheral blood, bronchial biopsies, and bronchoalveolar lavage [21-24]. In contrast, there is significant evidence supporting Th1-type inflammation in asthma from the increased levels of IFN-γ in the blood and induced sputum [25]. The latter may be a response that modulates the Th2 type inflammation through an epithelial-derived nitric oxide mediated pathway [26]. Furthermore, additional inflammatory cytokines such as IL-1β and TNF-α are derived primarily from macrophages (not Th1 or Th2-derived) and also play a significant role in asthma inflammation. Asthma is clearly a heterogeneous disease in onset, severity and response to therapy, and this observation may be, in part, due to the heterogeneous nature of the inflammation. While stimulation with cytomix and IL-13 are not meant to recapitulate Th1 and Th2 inflammatory responses, they do represent different features of inflammation.

An important feature of exhaled NO is the significant variability reported within a group of clinically similar individuals (e.g., healthy controls, asthma, cystic fibrosis, etc.). As with most biological signals, exhaled NO demonstrates a log normal distribution. At a constant exhalation flow of 50 ml/s (American Thoracic Society guidelines), the geometric mean value in healthy children (age 4–17 years) is 9.7 ppb, but the upper end of the 95% confidence interval is 25.2 ppb [27]. Similarly large ranges in healthy adults and clinically similar groups of subjects with asthma are invariably reported. These findings strongly suggest that our knowledge of the underlying source and determinants of exhaled NO remain crude. Our results of NO gas phase release from epithelial cells suggests the predominant type of inflammation and the anatomical source of the inflammation may be contributing factors to the variability of NO in the exhaled breath. For example, elevated Th2-type cytokine (IL-13) in large airways may lead to a significant increase in exhaled NO level; however, the same cytokine in the smaller airways may have little effect on exhaled NO.

Our *in vitro *system consisting of the airway epithelium for direct NO gas phase measurement enhances our understanding of the cellular-based mechanisms that affect exhaled NO. Our results demonstrate that NO gas phase release from SAECs and A549 is minimal at baseline, but is increased by cytomix reaching a rapid peak 10 hours following stimulation. In contrast, IL-13 does not impact NO gas phase release in A549, but does increase NO production in SAECs much more slowly. The NO release is closely linked temporally and in magnitude with iNOS protein levels.

## Conclusion

Together with previous results from NHBEs, we conclude that the lung epithelium can produce and release NO into the gas phase, and is the likely source of NO in the exhaled breath. However, the dynamics and magnitude depend strongly on the inflammatory stimulus and the anatomical location, which may contribute to the intersubject variability of the exhaled NO signal.

## Competing interests

The authors declare that they have no competing interests.

## Authors' contributions

JJ designed, planned, and performed all of the experiments, and wrote the manuscript; NKM assisted in SAEC culture; VS assisted in setting up the gas phase NO measurement system and analyzing the NO signal; and SCG provided overall guidance for the study, assisted in the experimental design, analysis and interpretation of the data, and writing of the manuscript. All authors have read and approved the final manuscript.
